# Diseases of the Urinary Organs: A Compendium of Their Diagnosis, Pathology, and Treatment

**Published:** 1859-01

**Authors:** 


ART. V.—*Diseases of the Urinary Organs. A Compendium of their Diagnosis, Pathology, and Treatment.* By WILLIAM WALLACE MORLAND, M.D., Fellow of the Massachusetts Medical Society, Member of the Boston Society for Medical Improvement, One of the Attending Surgeons at the Central Office of the Boston Dispensary, Corresponding Member of the Glasgow Medico-Chirurgical Society. With illustrations. Philadelphia: Blanchard & Lee, 1858. Octavo, pp. 579.

THIS publication, as we are told in the preface, is mainly composed of the substance of two essays, to which prizes were awarded by the Boylston Medical Committee, in the years 1855 and 1857. It is composed of two parts, the first of which, devoted to diagnosis, contains 128 pages, the second, devoted to pathology and treatment, contains 380 pages, and an appendix of 50 pages is attached, in which are collected thirty-eight alphabetically arranged paragraphs that were not incorporated in the text. The arrangement of this volume is altogether such as to render it a very difficult task to say what it contains and what it does not. The subjects are not completed in the same article, and the reader must search here and there to see how fully or how meagerly they have been treated of.

In the beginning of Part I., in the first chapter, the urinary organs are thus enumerated:—

"The urinary organs are the kidneys, which are true glands; the ureters, which are the first conduits for the transmission of the urine, as secreted; the bladder, which is its receptacle for a certain time; and the urethra, the second conduit for the urine when ready for excretion.

"The supra-renal capsules, although not classed with the above, will be considered in connection with them, being related by contiguity, and occasionally involved in the same diseases."

Most persons, at the present day, will not be disposed to regard the kidneys as true glands, if by the term *true glands*, as we must suppose, it is intended to convey the idea that they are veritable secretory organs, organs elaborating from the circulating fluid what did not previously exist therein. Evidently they cannot now be looked upon as other than excretory, as destined to eliminate from the blood effete matters; they secrete nothing. Any one holding other views of their functions must be exposed to make most egregious errors in treating of the pathology of the urinary organs. It is gratifying, therefore, to notice in some prefatory remarks upon the functions of the kidneys, in the third chapter, that they are declared to be "depurators of the blood, and to remove therefrom the unassimilated, superfluous, and effete albuminous principles." In justice to Mr. Morland it must be noticed, therefore, that by the term *true glands*, he does not wish to convey the idea usually attached to these words.

The prostate, as will be remarked by every one, is omitted in the list of organs treated of in this volume. It is never mentioned throughout, except to state that its enlargement may originate that condition of the bladder termed sacculated; that in extracting the calculus in the operation of lithotomy, it is sometimes lacerated; and in a few lines a case is related showing that when enlarged it may give rise to some symptoms deceptively resembling those of stricture of the urethra. Now, though far from partaking of Home's views as to the importance of this organ, yet we cannot hesitate to declare that a work upon the urinary organs, in which it is so entirely neglected, is exceedingly defective. What renders this neglect in regard to the prostate more extraordinary, is the attention paid to the supra-renal capsules. In the preface the author expressly states that he "strictly confines himself to those diseases arising in, or especially manifested by the organs in which the urine is elaborated, temporarily retained, and the passages through which it flows." Now with either the elaboration, the reception, or the conduction of the urine, the supra-renal capsules have nothing to do; in disorders of these functions they are never thought of; while, on the contrary, the prostate is encountered, and comes into consideration, in a very great number of the cases. It is really a fault of commission to have treated of the supra-renal capsules, as it is one of omission not to have treated of the prostate.

The remainder of this first chapter contains nothing remarkable, nor does the second, which treats of affections of the supra-renal capsules; this, however, is to be expected, for it was written, as we are informed in a note, before attention had been drawn to them by the investigations of Dr. Addison.

Chapter III. is upon diseases of the kidney. The enumeration of their diseases as adopted by Dr. George Johnson, in his well-known work on this subject, is followed with, as it is said, certain modifications of the arrangement. In his chapter upon suppurative nephritis, Dr. Johnson gives the history of those forms of renal disease which are attended with the secretion of pus, and in doing this, for the sake of convenience, divides the subject in the following manner:—

I.—Suppurative nephritis from morbid conditions of the blood.
II.—Nepritis from external violence.
III.—Nephritis from retention of urine.
IV.—Nephritis from calculi in the kidney.

Pyelitis is considered, by Dr. Johnson, under the head of retention of urine, as one of the most frequent consequences of the consequent dilatation of the kidney in inflammation of the mucous membrane of the pelvis, or pyelitis, as Rayer terms it. The certain modifications made by Dr. Morland consist simply in separating more distinctly these several subjects, without changing their order.

The text is illustrated by figures, which are all the same as those in Dr. Johnson's work, with two exceptions, the fifth and sixth, illustrating hemorrhage in the Malpighian capsules compressing the tufts, and a tube containing some yellow granules, the remains of extravasated blood. With the numerous facts and observations that have been made known since the time of the publication of Dr. Johnson's work, (1852,) Dr. Morland shows himself to be familiar, and he is always careful to state his authority. In doing this, however, he generally gives but the name of the person, without adding the treatise, and the exact page where the citation is to be found recorded; this is an omission of some importance, and one which his readers must necessarily regret.

Chapter IV. is upon diseases of the ureters, not by any means a fruitful subject, at least in an exclusively diagnostic point of view. As it is said: "The diagnosis is not usually difficult, particularly if there have been even slight symptoms of obstruction," (p. 81.) When, however, there are no such symptoms, it is exceeding difficult. In a case that came under our own notice, where there had not been at any period any lumbar pains, either spontaneous or excited by pressure, nor any mucous or purulent deposits in the urine, the usual symptoms of this affection, the patient was treated as laboring under an abscess of the spleen by one of the most distinguished surgeons in the world, if not the most distinguished, and the autopsy revealed that she had been laboring under obstructed ureter, and its local consequences.

The remaining two chapters of this first part, the fifth and the sixth, treat respectively of the diseases of the bladder and of the urethra. Here, as in the other chapters of this part, the task of the author is an embarrassing one. We scarcely know whether he may be said to have done it well or not. It is certainly a very awkward arrangement, both for the reader and for the author himself, this adopted by Dr. Morland, of collecting all the diagnostic symptoms of all the different diseases in a separate portion of his work. The text of these chapters is illustrated by figures, all of which are the same as those in Dr. Gross's work on the same subject, with the exception of the thirteenth, representing a calculus formed upon a hair-pin.

The first part ends with the following paragraphs:—

"We have thus passed in review the diagnostic elements peculiar to each affection of the urinary organs. When it is remembered that many large volumes have been written upon these diseases, and several even upon the separate one, it will be evident that he who essays to gather within a few pages the choice treasures so widely diffused, must roam far and tarry long—and yet be only a gleaner in the rich field. Our course has been to linger amid the weightiest sheaves. They are the freshest, and of undoubted quality.

"While preparing a digest of what is known relatively to the diagnosis of these important maladies, we have met with several practical illustrations of the truths we have endeavored to present. Scarcely any department of our art has had so many zealous and distinguished cultivators. Discovery after discovery has gleamed along their path, and the minute investigation of pathological changes has only been rivaled by the judiciousness of treatment, and the wonderful ingenuity displayed in the fabrication and use of instruments. No less industry has been manifested in the faithful study of the normal anatomy of the organs. The sagacity of the ancients was great, and has the respect of the present age for much accurate information, even diagnostically, which it accumulated under great disadvantages; the perseverance of the moderns, if it still continue like that of Bright, Christison, Bird, Prout, Johnson, Rayer, Civiale, and a host of others, will yet more nearly complete the lofty structure they have so far advanced—when there will be less occasion than now to say '*Multum adhuc restat operis, multumque restabit.*'"

The second part of the book, or that devoted to pathology and treatment, is divided likewise into six chapters, having the same headings as those in the first. This part, as the other, evidences quite extensive reading on the part of Dr. Morland; to use his own comparison, contained in the paragraph extracted, he has been an industrious gleaner. Gleaners, however, he might be told, are not to allow themselves to linger among the weightiest sheaves, though freshest and of undoubted quality; these belong to the masters of the field. We must not be misunderstood in what we say, for it would be very unjust on our part to convey the idea that Dr. Morland has appropriated improperly the labors of others; it is to his mode of expressing himself that we object. The first chapter opens as follows:—

"The term pathology has a wide significance. It properly comprises whatever enlightens us upon the nature and phenomena of disease; its materials are to be gathered during life and after death; by familiarity with the constitutional peculiarities of patients when in ordinary health, as well as when ill; their history, hereditary and personal; their habits; mental and bodily employment; their aspect and associations, are all pathological elements. To these the physician adds his deductions from healthy and morbid anatomy; physiological reasoning; comparisons between perfectly performed and deranged function; microscopical investigation, and the hints afforded by the action of remedies."

The last chapter thus ends:—

"To present a condensed view of the most important practical points suggested by the subject, has been the aim of the writer. The accumulation of new facts, new aspects of disorder, and remedial measures, tempts one to continual additions to such a *résumé* as has been given.

"There is scarcely a fresh treatise upon these topics but offers many interesting and essential views and hints; while medical periodicals, with almost every issue, inundate our fields of observation with novel cases, extended necroscopic examinations, plans of treatment, modified or original, and ingenious theories—which latter, if they subserve no other purpose, accustom the thoughtful mind to scrutinize closely and discriminate accurately.

"In the midst of so much floating material, there come to us treasures of information worth every effort to secure. The practitioner, as he surveys this constant stream, derived from such various sources, and seeks to appropriate whatever is truly valuable, has firm ground upon which to stand, in the imperishable productions of those brilliant and laborious investigators who have so satisfactorily explored the wide domain of these complex affections *consilio et manu.*"

In the appendix the various paragraphs, composed chiefly of extracts from the most recent journals, are arranged in the order given to the letters in the alphabet, and probably the same reason for this order exists in the one case as in the other.

We think that what we have said of this production, and the extracts we have made from it, are sufficient to enable our readers to judge of its character. The extracts we have made will enable them to judge also of the style of the author, which to our taste is not elegant, and, what is of more importance in a scientific work, it is far from being clear. We have been tempted to make another extract from page 342, to show what the author can do, in an endeavor to be witty, but we prefer to be generous.

We agree with Mr. Pott, "that he who thinks he can produce any benefit to society, needs not to be anxious about an apology for the publication of his ideas;" but this is hardly true of those who simply collect the ideas of others, and publish them all together, badly arranged, in a large mass.

